# Simulating water-nitrogen transport for winter wheat under drought-rewatering conditions in the North China

**DOI:** 10.3389/fpls.2026.1793353

**Published:** 2026-04-07

**Authors:** Yanbin Li, Aofeng He, Xuewen Gong, Shikai Gao, Qian Wang, Pengcheng He, Yihao Liu, Hao Li

**Affiliations:** 1 School of Water Conservancy, North China University of Water Resources and Electric Power, Zhengzhou, China; 2 Institute of Farmland Irrigation, Chinese Academy of Agricultural Sciences, Xinxiang, China

**Keywords:** ammonium nitrogen, HYDRUS, nitrate nitrogen, nitrogen transformation, water-nitrogen coupling, winter wheat

## Abstract

**Introduction:**

This study integrated field experiments with the HYDRUS-2D model to investigate the effects of water-nitrogen coupling on winter wheat yield and nitrogen transformation.

**Materials and methods:**

Field trials incorporated two irrigation regimes—conventional irrigation (75%–100% of field capacity) and drought stress (50%–60% of field capacity)—alongside three nitrogen application rates (100, 200, and 300 kg/ha) applied during the jointing and filling stages. The primary objective was to evaluate the impact of drought and rewatering on the spatiotemporal distribution, transformation, and leaching of soil nitrogen, and to assess the HYDRUS-2D model’s applicability in simulating non-steady-state water-nitrogen dynamics.

**Results:**

The results indicated that the HYDRUS-2D model demonstrated high accuracy in simulating the spatiotemporal dynamics of ammonium and nitrate nitrogen, with coefficients of determination R2 exceeding 0.78 and both RMSE and MAE values below 5.97. Regarding nitrogen transformation, drought during the jointing stage significantly inhibited nitrification, leading to the accumulation of ammonium nitrogen in the 0–40 cm soil layer. Upon rewatering, the rapid conversion of ammonium to nitrate nitrogen enhanced leaching into deeper soil layers (40–100 cm). Conversely, drought during the filling stage had a minimal impact on nitrogen transformation and posed a comparatively lower risk of nitrate leaching following rewatering. Finally, nitrogen application significantly influenced crop yield; moderate application (200 kg/ha) combined with adequate irrigation maximized winter wheat yield, whereas excessive application resulted in nitrogen loss and yield reduction.

**Discussion:**

These findings validate the model’s predictive capability and provide theoretical insights for optimizing water and nitrogen management to enhance crop yield and mitigate environmental pollution under water resource constraints.

## 1 Introduction

In the context of global food security and environmental protection, optimizing agricultural water and nitrogen management to achieve high crop yields and efficient resource utilization has emerged as a central challenge for sustainable agricultural development (Liu et al., 2021). Water and nitrogen are key limiting factors for crop growth, and their synergistic interactions exert a profound influence on soil biogeochemical cycles, crop productivity, and yield potential (Karandish and Šimůnek, 2017; Zaman et al., 2025). However, excessive irrigation and fertilization practices, driven by the goal of maximizing yields, have resulted in a range of severe environmental consequences. For example, long-term over-application of nitrogen in the North China Plain has led to significant nitrate contamination of groundwater (Liu et al., 2025; Wang et al., 2017), and agricultural nonpoint source pollution has become a major contributor to eutrophication in water bodies (Akinnawo, 2023; Hussain et al., 2025). Therefore, the development of water-nitrogen management strategies that both safeguard food security and minimize negative environmental impacts is urgently needed (Wu et al., 2024; Zhang et al., 2024).

To address this challenge, researchers have made substantial progress in optimizing water-nitrogen management strategies through the integration of field experiments and numerical models (Wang et al., 2023b). In terms of modeling, studies utilizing numerical models such as HYDRUS have effectively quantified water and nitrogen migration patterns under various irrigation practices, including wide ridge-furrow irrigation (Wang et al., 2023b), drip irrigation (Morianou et al., 2023), and subsurface drip irrigation (Beggs et al., 2011). Furthermore, research has expanded beyond water and nitrogen to model multi-dimensional interactions, such as water-heat-nitrogen and water-salt-nitrogen interactions (Li et al., 2023; Liu et al., 2024; Yadav et al., 2020). Regarding mechanistic exploration, studies have shifted from focusing solely on physical transport to investigating biochemical processes, such as elucidating the response mechanisms of soil enzyme activities (e.g., urease, sucrase) to water-nitrogen management (Yang et al., 2023). These studies have revealed a significant positive correlation between enzyme activity, soil nitrate nitrogen content, and crop yield (Yang et al., 2023). Collectively, these studies provide robust scientific evidence for optimizing water and fertilizer management under steady-state or near-steady-state conditions.

However, existing research has primarily focused on sustained, steady, or near-steady water regulation conditions, with relatively less attention given to the increasing frequency of extreme climate events, particularly transient drought stress during critical growth stages (e.g., jointing and grain filling) and the subsequent rewatering process (Wu and Yang, 2024). The timing of drought, rather than its intensity, may have distinct effects on crop recovery and soil nitrogen cycling (Kundel et al., 2021). Research has demonstrated that the jointing stage, which is characterized by active vegetative and reproductive growth, plays a crucial role in determining spike number and final yield (Zheng et al., 2024). In contrast, drought during the grain filling stage mainly affects grain filling and thousand-grain weight (Sheng et al., 2022). These growth-stage-specific stresses result in different nitrogen transformation and leaching dynamics following rewatering. For instance, ammonium nitrogen accumulated during the drought period may be rapidly nitrified upon rewatering. If the crop’s root absorption capacity has not fully recovered, this can significantly increase the risk of nitrate nitrogen leaching (Winter et al., 2023). More importantly, the non-steady, high-gradient water-nitrogen transport processes driven by drought rewatering present a substantial challenge to the predictive capabilities of widely used numerical models such as HYDRUS-2D. Although the model performs well in simulating ammonium nitrogen transport, its accuracy in predicting nitrate nitrogen leaching, particularly in deeper soil layers and under significant water fluctuations, still requires rigorous evaluation and validation (Morianou et al., 2023; Wang et al., 2023b). Some studies have suggested that the model’s inadequate representation of deep biochemical processes, such as denitrification, is a major limitation in its ability to predict deep nitrate nitrogen transport (Li et al., 2025).

This study conducted a rigorously controlled field trial on winter wheat, quantifying how post-drought irrigation combined with nitrogen application during the two key growth stages of jointing and grain filling modulates the spatial distribution, transformation, and leaching risk of ammonium nitrogen (NH4+-N) and nitrate nitrogen (NO3--N) in the soil, and how these processes ultimately affect yield. We further evaluated the applicability of the HYDRUS-2D model in simulating unsteady water and nitrogen dynamics, focusing on predicting nitrate behavior in deeper soil layers. We hypothesized that the impact of post-drought irrigation depends on the growth stage, with different responses at the jointing and grain-filling stages. This study, combining field data from specific growth stages with process-based simulations, provides mechanistic support for developing repeatable, climate-adaptive water and nitrogen management strategies for winter wheat in the North China Plain.

## 2 Materials and methods

### 2.1 Experimental site

**Figure 1:**
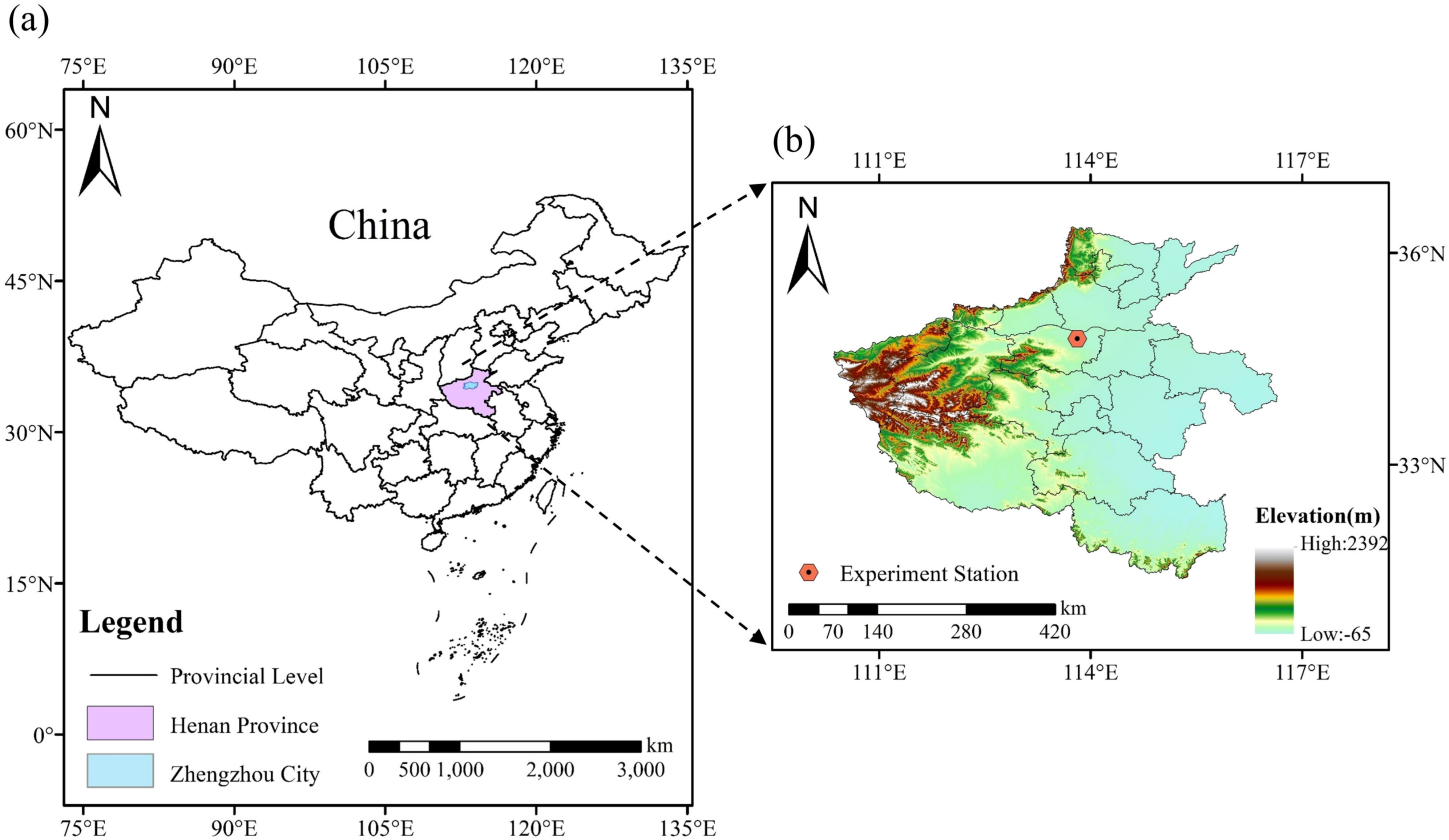
Research area. **(a)** illustrates the geographical location of the study area within the map of China, indicating the corresponding province and city; **(b)** depicts the geographical location of the study area within its respective province.

**Figure 2:**
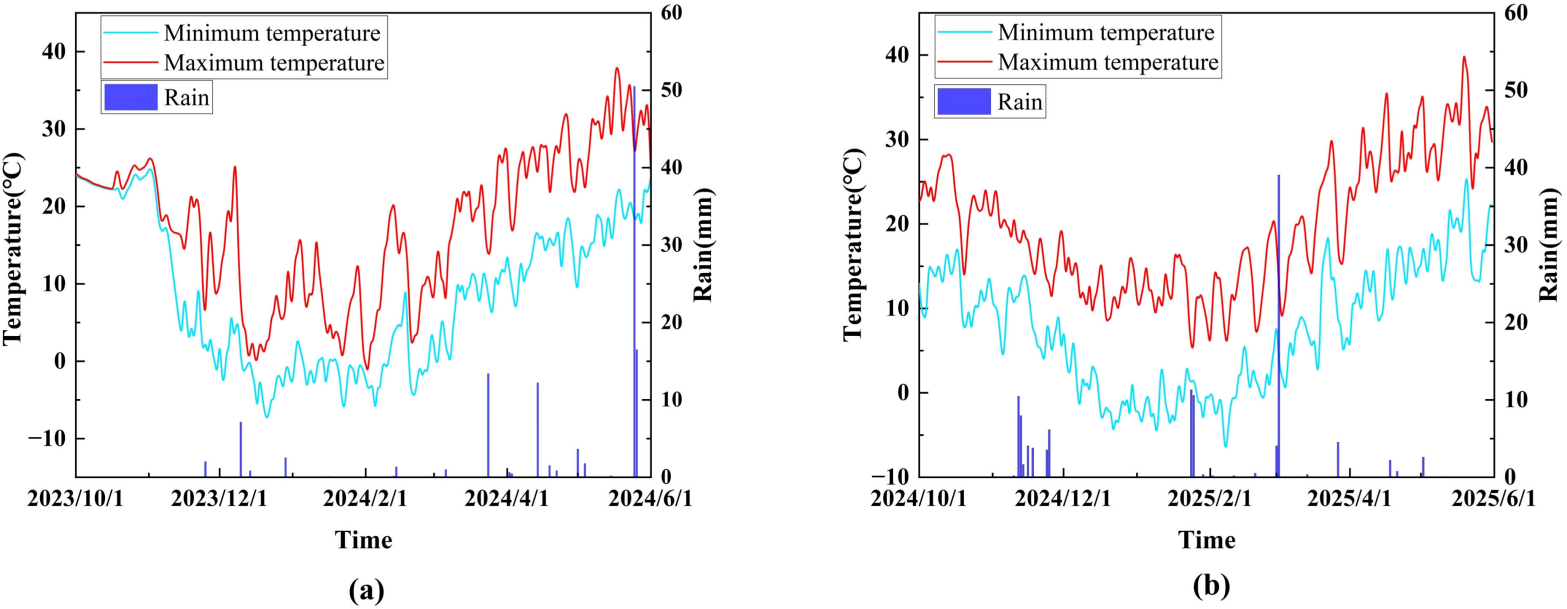
Meteorological data **(a)** 2023-2024, **(b)** 2024-2025.

The experiment was conducted at the Henan Provincial Key Laboratory of Water-Saving Agriculture, North China University of Water Resources and Electric Power (34°47’5.91”N, 113°47’20.15”E, **Figure 1**). The region is characterized by a semi-arid monsoon climate, with an average annual rainfall of 635.6 mm, an average annual temperature of 15.6 °C, and average annual evaporation of 1112.6 mm. Open field pits, each with dimensions of 3.0 m (length) × 3.0 m (width) × 1.0 m (depth), were used for the experiment, with a total of 27 pits arranged in three rows from east to west. Each pit was equipped with an automatic soil moisture monitoring system (ModelZL-06, INSENTEK Corp, Hangzhou, China) that updated moisture data every hour. The experimental setup also included a comprehensive irrigation and drainage system, and meteorological data were collected using an on-site weather station (RX3000, HOBO, Annapolis, MD, USA). **Figure 2** shows the meteorological data. The basic physicochemical properties of the soil layers from 0 to 100 cm are detailed in **Table 1**.

**Table 1:** Basic physical properties and physicochemical parameters of the soil.

Soil depth (cm)	Dry bulk weight of the soil (g/cm^3^)	Soil field capacity (V/V%)	Average content of total nitrogen (%)	Particle size composition (%)			Ammonium nitrogen (mg*·*kg*^−^*^1^)	Nitrate nitrogen (mg*·*kg*^−^*^1^)
				Clay	Sit	Sand		
0-20	1.46	34.6	0.04	4	45	51	9.52	7.95
20-40	1.47	35.2	0.05	5	45	50	7.85	7.64
40-60	1.47	35.2	0.03	5	45	50	8.46	6.35
60-80	1.46	35.4	0.02	3	45	52	6.89	6.12
80-100	1.48	35.4	0.01	3	45	52	6.31	6.48

### 2.2 Experimental design

The experiment utilized the winter wheat variety “Zhou mai22,”sown on November 4, 2023, and harvested on May 24, 2024, with a second planting season from October 15, 2024, to May 19, 2025. The experimental design focused on two critical growth stages: the jointing stage (P1) and the grain filling stage (P2). These stages were subjected to drought stress under two irrigation treatments: CK (normal moisture, maintaining soil moisture at 75%-100% of field capacity) and W1 (drought stress, maintaining soil moisture at 50%-60% of field capacity). Additionally, three nitrogen fertilization levels were tested: N1 (100 kg/ha, applying 200 g of urea), N2 (200 kg/ha, applying 400 g of urea), and N3 (300 kg/ha, applying 600 g of urea). Detailed processing combinations are shown in **Table 2**. The design consisted of 9 experimental treatments, each with three replicates, and employed a randomized block design. Phosphorus fertilizer (single superphosphate) and potassium fertilizer (potassium sulfate) were applied as base fertilizers before plowing, while nitrogen fertilizer was split into base and topdressing applications, with top-dressing applied during the jointing stage. Pest and disease control followed standard field management protocols.

**Table 2:** Water and fertilizer treatment combinations.

ID	Treatment	Water treatment	Soil field capacity (V/V%)	Nitrogen fertilizer (kg·ha^−1^)
1	CKN1	Well-watered	75% -100%	100
2	CKN2			200
3	CKN3			300
4	N1W1P1	Drought stress at the jointing stage	50%-60%	100
5	N2W1P1			200
6	N3W1P1			300
7	N1W1P2	Drought stress at the grain-filling stage	50%-60%	100
8	N2W1P2			200
9	N3W1P2			300

At the beginning of the experiment, soil moisture in all plots were adjusted to exceed 75% of field capacity. Irrigation amounts were then controlled based on the experimental treatment design. Following drought stress, the rewatering process aimed to re-store soil moisture to 80-85% of field capacity.

### 2.3 Measurements

#### 2.3.1 Soil water content

Soil moisture content was continuously monitored using an automated soil moisture monitoring system (ModelZL-06, INSENTEK Corp, Hangzhou, China) that recorded data every hour. The sensor was installed at the center of each plot at a depth of 100 cm. Prior to planting winter wheat, soil samples were collected using a soil auger to measure moisture content at different depths (0-100 cm) in 20 cm intervals. The soil samples were then oven-dried at 105 °C to a constant weight, and moisture content was calculated to calibrate the instrument data.

#### 2.3.2 Soil nitrogen content

Soil ammonium nitrogen (NH_4_^+^-N), nitrate nitrogen (NO_3_^−^-N), and total nitrogen content were measured during the jointing and grain filling stages. Sampling was conducted prior to drought treatment, immediately after drought treatment, and one week following rewatering. Soil samples were collected at depths of 0-100 cm, with specific sampling points at 10, 20, 30, 40, 60, 80, and 100 cm. The samples were air-dried, ground, and sieved before being analyzed using the following methods:

Ammonium and Nitrate Nitrogen Analysis: A 10.00 g fresh soil sample was combined with 100 mL of 2 mol L^−1^ KCl solution and shaken at 25 °C for 1 h at 220 rpm on a mechanical shaker (Model ZWY-2102C, Shanghai Zhicheng Analytical Instrument Manufacturing Co., Ltd., China). The suspension was then filtered through Whatman Grade 42 ashless quantitative filter paper (nominal particle retention ~2.5 μm) to obtain a particle-free filtrate, which was analyzed within 24 h using an AA3 continuous/segmented flow analyzer (SEAL Analytical GmbH, Norderstedt, Germany). The mass fraction of inorganic nitrogen in soil was calculated using Equation 1.

(1) $$\omega \left(N\right)=\frac{C\times 100\times 0.001}{m}\times 1000$$

where ω_n_ represents the mass fraction of a specific inorganic nitrogen in the soil, expressed in (mg·kg^−1^);c represents the measured concentration, expressed in (mg·L^−1^);100 represents the volume of potassium chloride used, in(ml);10^−3^ represents the conversion factor to convert mass (m) into the appropriate units; 1000 represents the conversion factor to express the nitrogen content per kg of soil; m represents the soil mass, in grams (g).

#### 2.3.3 Yield and its component

At crop maturity, a 1 m^2^ harvest area is selected from each plot. Plants within the harvest area are manually trashed, and grains are collected. The grain quantity is determined by counting the grains in the threshed sample. Grain weight and thousand-grain weight are measured using an electronic analytical balance (Model DTY-B6000, HUAZHI (Fujian) Electronic Technology Co., Ltd, Putian, China, accuracy: 0.01 g). The grains yield is calculated based on the grain weight per square meter of harvested area and converted to kilograms per hectare(kg·ha^−1^).

### 2.4 Model construction

The accuracy of the model was evaluated using the Mean Absolute Error (MAE), Root Mean Square Error (RMSE), Coefficient of Determination (R²), and Nash-Sutcliffe Efficiency (NSE).

#### 2.4.1 Governing equation

In HYDRUS, the core simulation module focuses on water movement. It assumes that the effects of air on water flow are negligible and utilizes the Richards equation to describe water flow in both saturated and unsaturated zones, as shown in Equation 2 (Schaap and van Genuchten, 2006):

(2) $$\frac{\partial \theta }{\partial t}=\frac{\partial }{\partial Z}\left[K\left(h\right)\left(\frac{\partial h}{\partial Z}+1\right)\right]-S\left(Z,t\right)$$

where θ represents the soil volumetric water content (L^3^· L^−3^), t denotes time (T), h is the soil water potential head (L), Z is the vertical spatial coordinate (L, positive downward), K is the hydraulic conductivity function (L· T^−1^), and S is the root water uptake rate (L^3^· L^−3^· T^−1^).

This study employed the van Genuchten -Mualem model to describe the relationships between hydraulic conductivity (*K*), water potential head (h), and volumetric water content (*θ*) in winter wheat soil, as expressed in Equations 3–5:

(3) $$\theta \left(h\right)=\theta_{r}+\frac{\theta_{s}-\theta_{r}}{{\left[1+{\left(\alpha h\right)}^{n}\right]}^{m}},h\leq 0$$

(4) $$K\left(h\right)=K_{s}S_{e}^{'}{\left(1-{\left(1-S_{e}^{1/m}\right)}^{m}\right)}^{2}$$

(5) $$m=1-\frac{1}{n}\text{and }S_{e}=\frac{\theta -\theta_{r}}{\theta_{s}-\theta_{r}}$$

where *θ_r_* and θ_s_ represent the residual and saturated water contents (L^3^· L^−3^), respectively; α (L^−1^), m and n are fitting parameters for the soil water retention curve; l is the pore connectivity parameter (set to 0.5); *K_s_* is the saturated hydraulic conductivity (L· T^−1^); and S_e_ denotes the effective saturation.

#### 2.4.2 Nitrogen transport and transformation

The convection-dispersion governing partial differential equations for urea-N, NH_4_^+^-N, and NO_3_^–^-N transport and transformation in unsaturated soil are given in Equations 6–8, respectively (Simunek et al., 1996):

(6) $$\frac{\partial {\theta c}_{w,1}}{\partial t}=\frac{\partial }{\partial Z}\left({\theta D}_{1}\frac{\partial c_{w,1}}{\partial Z}\right)-\frac{\partial {qc}_{w,1}}{\partial Z}{-\mu }_{w,1}^{'}{\mathrm{\theta c}}_{w,1}\;$$

(7) $$\frac{\partial \theta c_{w,2}}{\partial t}+\rho \frac{\partial c_{s,2}}{\partial t}=\frac{\partial }{\partial Z}\left(\theta D_{2}\frac{\partial c_{w,2}}{\partial Z}\right)-\frac{\partial qc_{w,2}}{\partial Z}+\mu_{w,1}^{'}\theta c_{w,1}-\left(\mu_{w,2}+\mu_{w,2}^{'}\right)\theta c_{w,2}-\left(\mu_{w,2}+\mu_{s,2}^{'}\right)\rho c_{s,2}+\gamma_{w,2}\theta +\gamma_{s,2}\rho -S\left(Z,t\right)c_{r,2}$$

(8) $$\frac{\partial {\theta c}_{w,3}}{\partial t}=\frac{\partial }{\partial Z}\left({\theta D}_{3}\frac{\partial c_{w,3}}{\partial Z}\right)-\frac{\partial {qc}_{w,3}}{\partial Z}{+\mu }_{w,2}^{'}{\theta c}_{w,2}{+\mu }_{s,2}^{'}{\rho c}_{s,2}{-\mu }_{w,3}{\theta c}_{w,3}{-\mu }_{s,3}{\rho c}_{s,3}{-S(Z,t)c}_{r,3}$$

where subscripts 1, 2, and 3 denote the nitrogen forms: urea, ammonium, and nitrate, respectively; *c_x_, c_s_*, and *c_r_* represent the nitrogen concentrations in the liquid phase (M· L^−1^), solid phase (M· M^−1^), and root water uptake (M· L^−3^), respectively; μ is the soil bulk density (M· L^−3^); D is the dispersion coefficient (L^2^·T^−1^); q is the volumetric water flux (L·T^−1^); μ represents the first-order nitrogen transformation rate constants (T^−1^); γ denotes the first-order rate constants linking transformations between urea, ammonium, and nitrate (T^−1^); and κ represents the zero-order nitrogen transformation constants (M· L^−3^ ·T^−1^).The diffusion co-efficient of nitrogen in water was estimated using:

(8) $$\theta D=D_{L}\left|q\right|+\theta D_{\tau }^{w}$$

where *θD* w_o_ is the molecular diffusion coefficient in free water (L^2^·T^−1^); τ is the tortuosity factor for the liquid phase; and *D_l_* is the longitudinal dispersity (L). The final terms in Equations 7, 8 represent nitrogen uptake via root water and passive root absorption, respectively. Since the adsorption of urea and nitrate by wheat soil is minimal, only their transport and transformation in the liquid phase were simulated.

#### 2.4.3 Root water uptake

Root water uptake in the model was simulated using the Feddes model, as given in Equation 9 (Feddes et al., 1982):

(9) $$S\left(\begin{array}{c} h,z,t \end{array}\right)=\alpha \left(\begin{array}{c} h \end{array}\right)S_{\max}$$

The maximum root water uptake rate is defined by Equation 10.

(10) $$S_{\max}=\beta (z,t)T_{p}(t)$$

where α(*h*) is the stress response function (ranging from 0.0 to 1.0), representing the effect of soil pressure head (or volumetric water content) on root water uptake; *T_p_(t)* is the potential root water uptake rate (d^−1^), which is related to the potential transpiration rate.

This study employed the Feddes model, which describes wheat root water uptake using a piecewise function *α(h)* based on four critical soil pressure head values (*h_4_< h_3_< h_2_< h_1_*). This study employed the Feddes model, which describes root water uptake under water stress conditions, and the water stress response function is expressed by Equation 11.

(11) $$\alpha \left(h\right)=\{\begin{array}{c} \frac{h-h_{4}}{h_{3}-h_{4}}\;\;\;\;\;\;\;\;h_{3}>h>h_{4}\\ \;\;\;\;\;1\;\;\;\;\;\;\;\;\;\;\;\;h_{2}\geq h\geq h_{3}\\ \frac{h-h_{4}}{h_{3}-h_{4}}\;\;\;\;\;\;\;\;h_{1}>h>h_{2}\\ \;\;\;\;\;0\;\;\;\;\;\;\;h\leq h_{4}\;or\;h\geq h_{1} \end{array}$$

The four critical pressure heads used in the simulation were *h_1_* = -1 cm, *h_2_* = -500 cm, *h_3_* = -900 cm, and *h_4_* = -1600 cm.

Given the low salinity of the experimental soil, salt stress was considered negligible. Therefore, the actual transpiration rate of winter wheat was calculated using Equation 12:

(12) $$ET_{a}=\int_{L_{R}}S\left(h,z,t\right)dz=T_{p}(t)\int_{L_{R}}\alpha (h)\beta (z,t)dz$$

where *L_r_* represents the root distribution zone, which is described by the root growth model embedded within HYDRUS-2D (Equation 13):

(13) $$L_{R}(t)=L_{m}f_{r}(t)$$

where *L_m_* represents the maximum root depth of wheat (cm), and *f_r_(t)* is a dimensionless root growth coefficient, estimated using the Verhulst-Pearl logistic growth function (Equation 14):

(14) $$\;f_{r}\left(t\right)\;=\frac{L_{0}}{L_{0}+\left(L_{m}-L_{0}\right)e^{-rt}}$$

where L_o_ represents the initial root length at the start of the simulation stage (cm); *R* is the root growth rate (day^−1^), determined through destructive sampling conducted twice during each winter wheat growth stage. During the simulation, *L_o_* was set to 1 cm for all treatments, while *L_m_* ranged from 50 to 55 cm, varying according to the degree of water and nitrogen deficit.

#### 2.4.4 Evapotranspiration model

Daily precipitation, mean air temperature, relative humidity, sunshine duration, and wind speed were recorded via an on-site weather station (RX3000, HOBO, Annapolis, MD, USA). Reference evapotranspiration (*ET_O_*, mm**·**day^-1^) was calculated using the Penman-Monteith formula (Equation 15):

(15) $$ET_{O}=\frac{0.408\Delta \left(R_{n}-G\right)\gamma \frac{900}{T+273}u_{2}\left(e_{S}-e_{a}\right)}{\Delta +\gamma \left(1+0.34u_{2}\right)}$$

Where *Rn* represents the net radiation at the crop surface (MJ·m^−2^·day^−1^), *G* represents the soil heat flux (MJ·m^−2^·day^−1^), *T* represents the mean daily air temperature (°C), *u_2_* represents the wind speed at a height of 2 meters (m·S^-1^), (*e_S_ - e_a_*) represents the vapor pressure deficit (kPa), Δ represents the slope of the saturation vapor pressure curve at temperature T (kPa·°C^−1^), and *γ* represents the psychrometric constant (kPa·°C^−1^).

The potential crop evapotranspiration (*ETc*) was determined using the single crop coefficient method, and *ETc* was further partitioned into potential transpiration (*T_P_*) and soil evaporation (*E_P_*) using Equations 16 and 17, respectively:

(16) $$ET_{c}=k_{c}ET_{O}$$

(17) $$ET_{c}=T_{P}+E_{P}=ET_{c}\left(1-e^{-0.6LAI}\right)+ET_{c}e^{-0.6LAI}$$

where *k_c_* is the actual crop coefficient determined following (Ko et al., 2009), and *LAI* is the leaf area index.

### 2.5 Model parameter

The model parameters were derived from field measurements conducted in 2023-2024, encompassing soil moisture, soil particle composition, bulk density, ammonium nitrogen, nitrate nitrogen in winter wheat treatments. The initial hydraulic characteristic parameters were predicted using model data. The final hydraulic characteristic parameters and solute transport parameters were derived through calculation by the inversion module built into HYDRUS. These parameters are elaborated in **Tables 3**, **4**. After completing the calibration of HYDRUS-2D, we performed a simplified sensitivity analysis of nitrogen reaction parameters using a one-at-a-time (OAT) approach. In this method, only one parameter is perturbed at a time while all other parameters are held at their baseline values, and the corresponding model output response is evaluated. Following the commonly used HYDRUS nitrogen reaction chain formulation (urea → NH_4_^+^ → NO_3_^−^), three reaction parameters were selected for the screening test: μ_w,1_ (the first-order term associated with urea), μ_w,2_ (the chain-reaction term representing nitrification), and μ_w,3_ (the first-order removal term for NO_3_^−^). Each parameter was perturbed by ±20%, and the peak NO_3_^−^-N concentration in the deep soil layer (40–100 cm) was used as the sensitivity metric. The relative change in the deep-layer NO_3_^−^-N peak concentration was quantified using Equation 18:

**Table 3:** Parameters of soil hydraulic properties.

Depth/(cm)	θ_r_	θ_s_	α(cm^-1^)	n	K_s_(cm·day^-1^)
0-20	0.031	0.410	0.0136	1.483	55.54
20-60	0.032	0.407	0.0125	1.490	48.82
60-100	0.029	0.413	0.0160	1.465	62.31

**Table 4:** Soil solves transport parameters.

Soil depth (cm)	μ_w,1_ (day^-1^)	μ_w,2_ (day^-1^)	μ^’w,2^ (day^-1^)	μ^’s,2^ (day^-1^)	μ_w,3_ (day^-1^)	μ_s,3_ (day^-1^)	γ_w,2_ (μg · cm^-3^ · day^-1^)	γ_s,2_ (μg · cm^-3^ · day^-1^)
0-20	0.56	0.023	0.25	0.25	0.04	0.04	5*10^-5^	5*10^-5^
20-60	0.55	0.030	0.26	0.26	0.03	0.03	5*10^-6^	5*10^-6^
60-100	0.57	0.37	0.36	0.36	0.04	0.04	3*10^-6^	3*10^-6^

(18) $$\text{Δ}\left(\%\right)=\left(Y_{i}-Y_{0}\right)/Y_{0}\times 100$$

where Δ(%) is the percent change in the deep-layer (40–100 cm) NO_3_^−^-N peak concentration relative to the baseline, *Y_o_* is the baseline peak value, and *Y_o_* is the peak value obtained under the *i*-tch parameter perturbation scenario.

A normalized sensitivity index was then calculated using Equation 19:

(19) $$SI=\frac{\left(Y_{i}-Y_{0}\right)/Y_{0}}{\left(p_{i}-p_{0}\right)/p_{0}}=\text{Δ}\left(\%\right)/20\%$$

where *SI* is the normalized sensitivity index, p_o_ is the baseline value of the perturbed parameter, p_o_ is the parameter value in the i-th perturbation scenario (*p_i_ = p_o_ × (1 ± 0.20*)), and *(p_i_ − p_o_)/p_o_* is the relative parameter change (± 20% in this study).

### 2.6 Initial condition and boundary condition

#### 2.6.1 Initial condition

**Figure 3:**
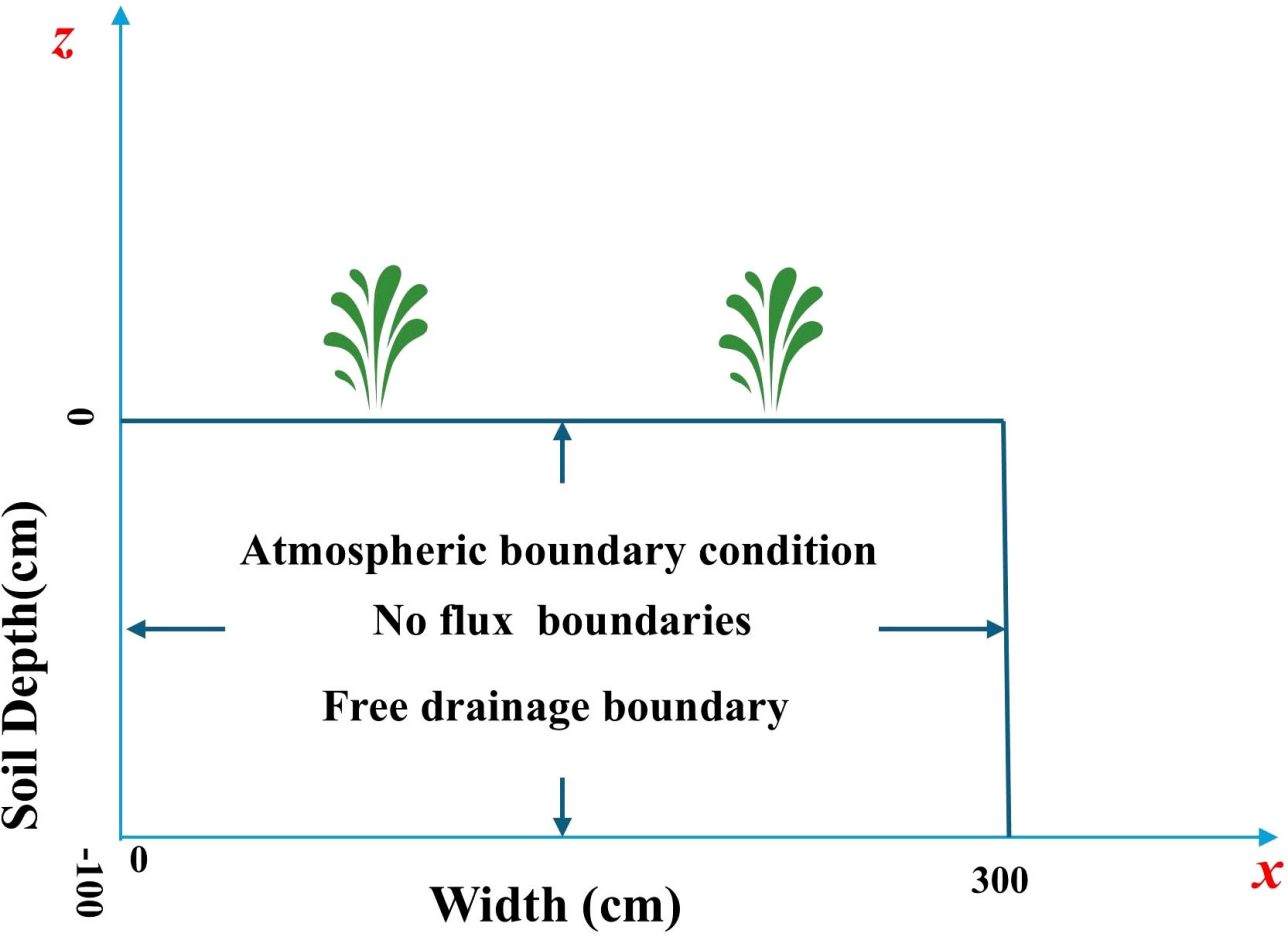
The geometric structure and boundary conditions of the model.

To simulate the coupled transport of water and nitrogen in the winter wheat field, a 1-meter-deep soil profile (**Figure 3**) was selected to represent the soil column in the experimental lysimeters. Since the groundwater table remained consistently below 1 m throughout the experimental period, this profile depth was considered sufficient to capture the primary vertical water movement within the root zone. The simulation period began on the day of topdressing at the jointing stage. The initial soil water content and initial solute concentration in the simulation domain were specified by Equations 20 and 21, respectively:

(20) $$\theta (r,z,t)=\theta_{k}(0\leq r\leq L,0\leq z\leq H,t=0)$$

(21) $$c(r,z,t)=c_{k}(0\leq r\leq L,0\leq z\leq H,t=0)$$

where *θ_o_* is the initial soil volumetric water content (cm^3^·cm^−3^);*C_k_* represents the initial concentration of nitrate nitrogen (or ammonium nitrogen) in the soil (mg·cm^−3^);*R* is the ra-dial coordinate *(*cm);z is the vertical coordinate, defined as positive downward (cm);*L* is the horizontal distance of the simulation domain, with 0 ≤ *L* ≤ 300 cm; H is the soil depth, representing the depth of the computational domain, with 0 ≤ *H* ≤ 100 cm.

#### 2.6.2 Boundary condition

For the simulation domain, the lateral (left and right) boundaries were defined as no flux boundaries. The lower boundary was set as a free drainage boundary, while the upper boundary was specified by atmospheric boundary conditions (**Figure 3**).

The hydraulic and solute transport boundary conditions applied in the model are summarized in Equation (22):

(22) $$\begin{array}{l} -K(h)\frac{\partial h}{\partial z}-K(h)=\sigma (t)(0\leq x\leq w,z=100,0<t<T)\\ -(\theta D_{\chi \chi }\frac{\partial c_{1}}{\partial x}+\theta D_{\chi Z}\frac{\partial c_{1}}{\partial x})+q_{x}c_{1}=q_{x}c_{-}(\theta \leq x\leq w,z=100,0<t<T)\\ -(\theta D_{zz}\frac{\partial c_{1}}{\partial z}+\theta D_{\chi Z}\frac{\partial c_{1}}{\partial x})+q_{z}c_{1}=q_{x}c_{-}(\theta \leq x\leq w,z=100,0<t<T) \end{array}$$

Where *c_o_* is the mass concentration of urea nitrogen in the fertilizer solution *(*mg·m^−3^); T is the duration for surface water to fully infiltrate the soil (*h*); *σ*(t) is the constant flux boundary condition at the irrigation point during the irrigation event, with a value of 3 cm·d^−1^.

### 2.7 Data processing

All experimental data were analyzed and organized using Excel 2021. A two-way analysis of variance (ANOVA) was performed to assess significant differences between the samples. Subsequently, all data were subjected to one-way ANOVA for significance testing using SPSS 27 (SPSS Inc., Chicago, Illinois, USA) with a significance threshold of *P<* 0.05. The data were also tested for normality using the Kolmogorov-Smirnov test. If the data followed a normal distribution, statistical analysis was conducted using ANOVA with a significant level of 5%. For data that did not follow a normal distribution, non-parametric tests were applied. All graphs were generated using Origin Pro 2024.

## 3 Results

### 3.1 Model calibration and parameter sensitivity analysis

**Figure 4:**
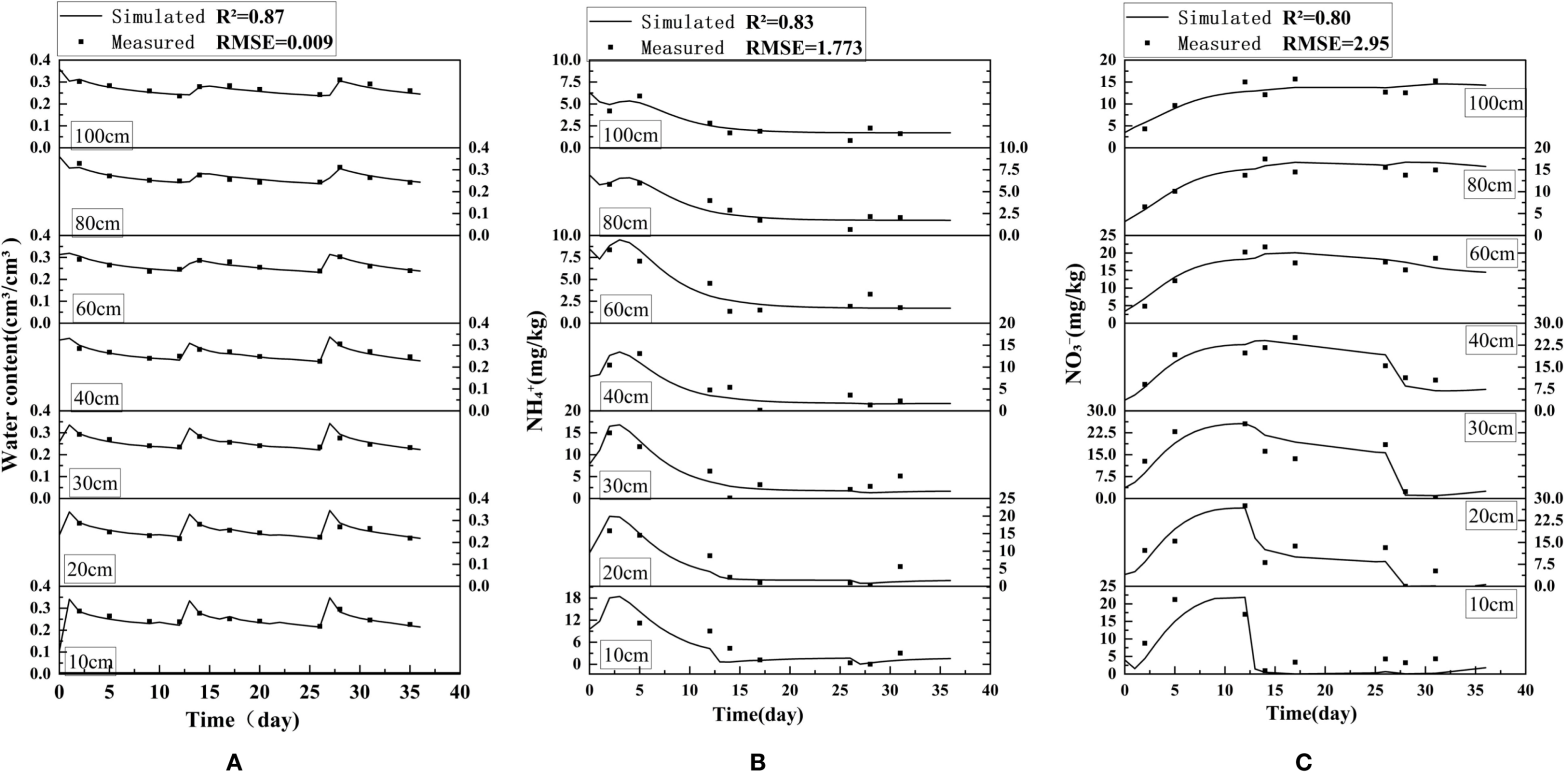
Comparison between measured and simulated values for **(a)** soil water content, **(b)** ammonium nitrogen, and **(c)** nitrate nitrogen at different soil depths.

The model was calibrated using field experimental data from 2023 to 2024, with data from the CKN2 treatment group selected for analysis. **Figure 4** presents a comparison between the measured and simulated values of soil water content, ammonium nitrogen, and nitrate nitrogen at different soil depths during the wheat growing season. The results show that the simulated soil moisture, ammonium nitrogen, and nitrate nitrogen concentrations all achieved high accuracy, with R^2^ values exceeding 0.80. The maximum RMSE for soil water content was 0.9%, indicating minimal simulation error. Similarly, the RMSE values for ammonium nitrogen (1.773 mg·kg^-1^) and nitrate nitrogen (2.95 mg·kg^-1^) were low, further confirming the model’s reliability in simulating solute dynamics. These outcomes demonstrate that the calibrated soil hydraulic parameters (**Table 3**) and solute transport and transformation parameters (**Table 4**) yield high-precision simulations with limited error.

**Table 5** show that, for the baseline treatment CKN2, the deep-layer (40–100 cm) NO_3_^−^-N peak was 24.20 mg·kg^−1^. Within the ±20% perturbation range, μw,3 exerted the largest influence on the peak: decreasing μw,3 by 20% increased the peak by 6.69%, whereas increasing μw,3 by 20% decreased the peak by 5.91%, yielding |*SI*|max = 0.34. The nitrification chain term μ^’w,2^ showed the second-largest impact, while the urea-associated first-order term μw,1 was comparatively less influential (|*SI*|max = 0.14). Overall, the sensitivity ranking was μ_w,3_ > μ^’w,2^ > μ_w,1_, indicating that, when the deep-layer peak is used as the evaluation criterion, the along-profile first-order removal strength of NO_3_^−^ (μ_w,3_) provides the dominant peak-attenuation control, followed by nitrification-driven production (μ^’w,2^), whereas the upstream urea-related conversion (μ_w,1_) is relatively insensitive under the conditions of this study.

**Table 5:** OAT sensitivity analysis of reaction parameters based on the deep layer(40-100cm) NO_3_^−^ peak.

Treatment	Base	μ_w_,1-20%	μ_w_,1 + 20%	μ_’w_,2-20%	μ_’w_,2 + 20%	μ_w_,3-20%	μ_w_,3 + 20%
Peak(mg/kg)	24.20	23.54	24.68	23.15	24.77	25.82	22.77
Δ(%)	0.00	-2.76	1.97	-4.33	2.36	6.69	-5.91
SI	0.00	0.14	0.01	0.22	0.12	-0.34	-0.30

### 3.2 Model validation

**Figure 5:**
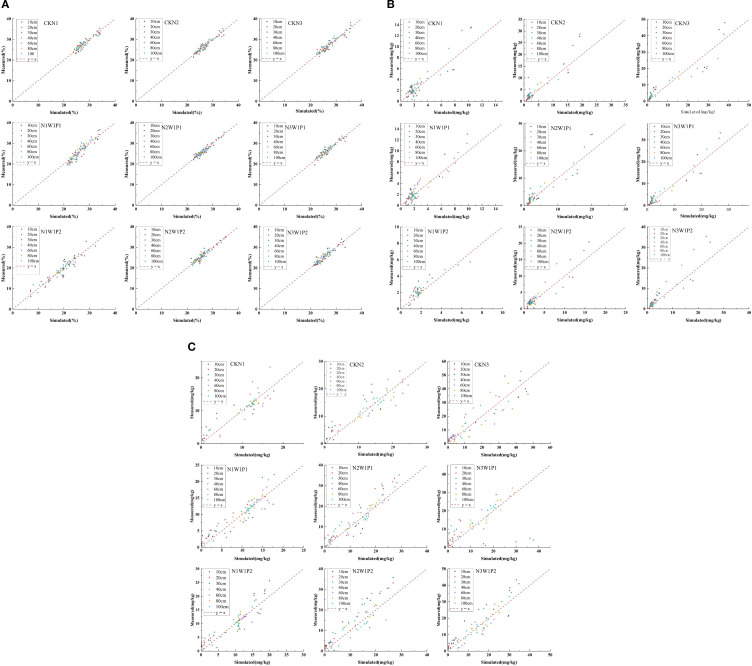
Fitting of water content, ammonium nitrogen, and nitrate nitrogen. **(A)** Fitting of Water content from 2024 to 2025. **(B)** Fitting of Ammonium Nitrogen content from 2024 to 2025. **(C)** Fitting of Nitrate Nitrogen content from 2024 to 2025.

During the period of 2024–2025, the model was applied to simulate various treatments, and the simulation results were compared against field experimental data to validate the calibrated soil hydraulic properties (**Table 3**) and solute transport parameters (**Table 4**). **Figure 5** present a comparison between the measured and predicted values of soil water content, ammonium nitrogen (NH_4_^+^-N), and nitrate nitrogen (NO_3_^−^-N) at various stages, from the winter wheat green-up stage to maturity. It can be observed that the revised soil hydraulic and solute transport transformation parameters demonstrate high accuracy (**Tables 6**, **7**). The model exhibits excellent simulation performance for soil nitrogen dynamics under varying water and nitrogen treatments. For ammonium nitrogen, the average R^2^ for all treatments exceeded 0.85, with RMSE and MAE values below 3.43 and 2.33, respectively, and NSE values surpassing 0.79. These results suggest that the model can effectively capture the adsorption, desorption, and transformation processes of ammonium nitrogen in the soil, with simulation accuracy remaining largely unaffected by water-nitrogen management strategies.

**Table 6:** Error analysis of measured and simulated soil volumetric water content, ammonium nitrogen, nitrate nitrogen values.

Treatment	Classification	R^2^	RMSE	NSE	MAE
CKN1	Water content	0.88	0.011	0.88	0.008
	NH_4_^+^	0.85	1.062	0.82	0.741
	NO_3_^−^	0.84	2.513	0.84	1.922
CKN2	Water content	0.90	0.009	0.90	0.007
	NH_4_^+^	0.89	2.323	0.84	1.358
	NO_3_^−^	0.86	3.134	0.83	2.493
CKN3	Water content	0.90	0.011	0.89	0.008
	NH_4_^+^	0.90	3.430	0.87	2.329
	NO_3_^−^	0.86	5.969	0.85	4.812
N1W1P1	Water content	0.88	0.015	0.86	0.011
	NH_4_^+^	0.88	0.933	0.86	0.701
	NO_3_^−^	0.86	2.050	0.85	1.632
N2W1P1	Water content	0.91	0.008	0.91	0.006
	NH_4_^+^	0.90	1.667	0.89	1.158
	NO_3_^−^	0.88	3.442	0.86	2.660
N3W1P1	Water content	0.90	0.009	0.90	0.007
	NH_4_^+^	0.92	1.971	0.91	1.521
	NO_3_^−^	0.90	4.308	0.87	3.503
N1W1P2	Water content	0.89	0.017	0.88	0.013
	NH_4_^+^	0.89	1.031	0.79	0.659
	NO_3_^−^	0.84	2.320	0.83	1.832
N2W1P2	Water content	0.89	0.010	0.89	0.008
	NH_4_^+^	0.88	1.407	0.86	0.890
	NO_3_^−^	0.84	4.450	0.78	3.642
N3W1P2	Water content	0.87	0.011	0.89	0.008
	NH_4_^+^	0.88	2.772	0.82	1.603
	NO_3_^−^	0.85	4.693	0.83	3.761

**Table 7:** Error analysis of vertical profile.

Treatment	Classification	Depth(cm)	R^2^	RMSE	NSE	MAE
N1W1P1	Water content	10	0.93	0.010	0.93	0.009
		20	0.90	0.011	0.89	0.009
		30	0.91	0.008	0.91	0.007
		40	0.89	0.008	0.89	0.006
		60	0.91	0.005	0.91	0.004
		80	0.89	0.006	0.89	0.005
		100	0.91	0.006	0.91	0.005
	NH_4_^+^	10	0.90	2.401	0.89	1.637
		20	0.94	2.082	0.92	1.262
		30	0.86	2.219	0.86	1.711
		40	0.93	1.233	0.92	1.053
		60	0.85	1.101	0.83	0.990
		80	0.85	0.946	0.77	0.759
		100	0.78	0.971	0.64	0.758
	NO_3_^−^	10	0.92	3.741	0.88	2.801
		20	0.94	4.284	0.86	3.450
		30	0.92	3.762	0.90	3.073
		40	0.95	4.862	0.69	4.388
		60	0.81	2.558	0.80	2.132
		80	0.84	1.933	0.80	1.490
		100	0.81	1.557	0.75	1.303

For nitrate nitrogen, the simulation accuracy is similarly high, with an average R^2^ exceeding 0.84 and an average NSE greater than 0.78. The RMSE and MAE values for nitrate nitrogen are slightly higher than those for ammonium nitrogen, primarily due to its greater mobility in the soil. The transport of nitrate nitrogen is influenced by multiple coupled factors, including water movement, crop uptake, and microbial transformations, which make its simulation more complex. Nevertheless, the model reliably captures the accumulation and leaching patterns of nitrate nitrogen under varying water-nitrogen treatments.

To further assess the model’s vertical simulation capability within the soil profile, the representative treatment N2W1P1 was selected, and the simulation accuracy at different soil depths was evaluated. The results indicate that the model demonstrated exceptionally high reliability across the entire 0-100 cm soil profile. The R^2^ values for ammonium and nitrate nitrogen at all depths exceeded 0.78, with RMSE and MAE values controlled below 4.85 and 4.38, respectively, and NSE values surpassing 0.64. Notably, in the 0-40 cm soil layer, where the crop root system is predominantly located, the model captured nitrogen dynamics with even greater accuracy.

**Table 6** presents an error analysis of the model, demonstrating a coefficient of determinization above 0.84 for each index, and indicating low mean absolute error (MAE) and root mean square error (RMSE) levels. The order of agreement between simulated and measured values was water content > ammonium nitrogen > nitrate nitrogen.

### 3.3 Transport water in soil profiles

**Figure 6:**
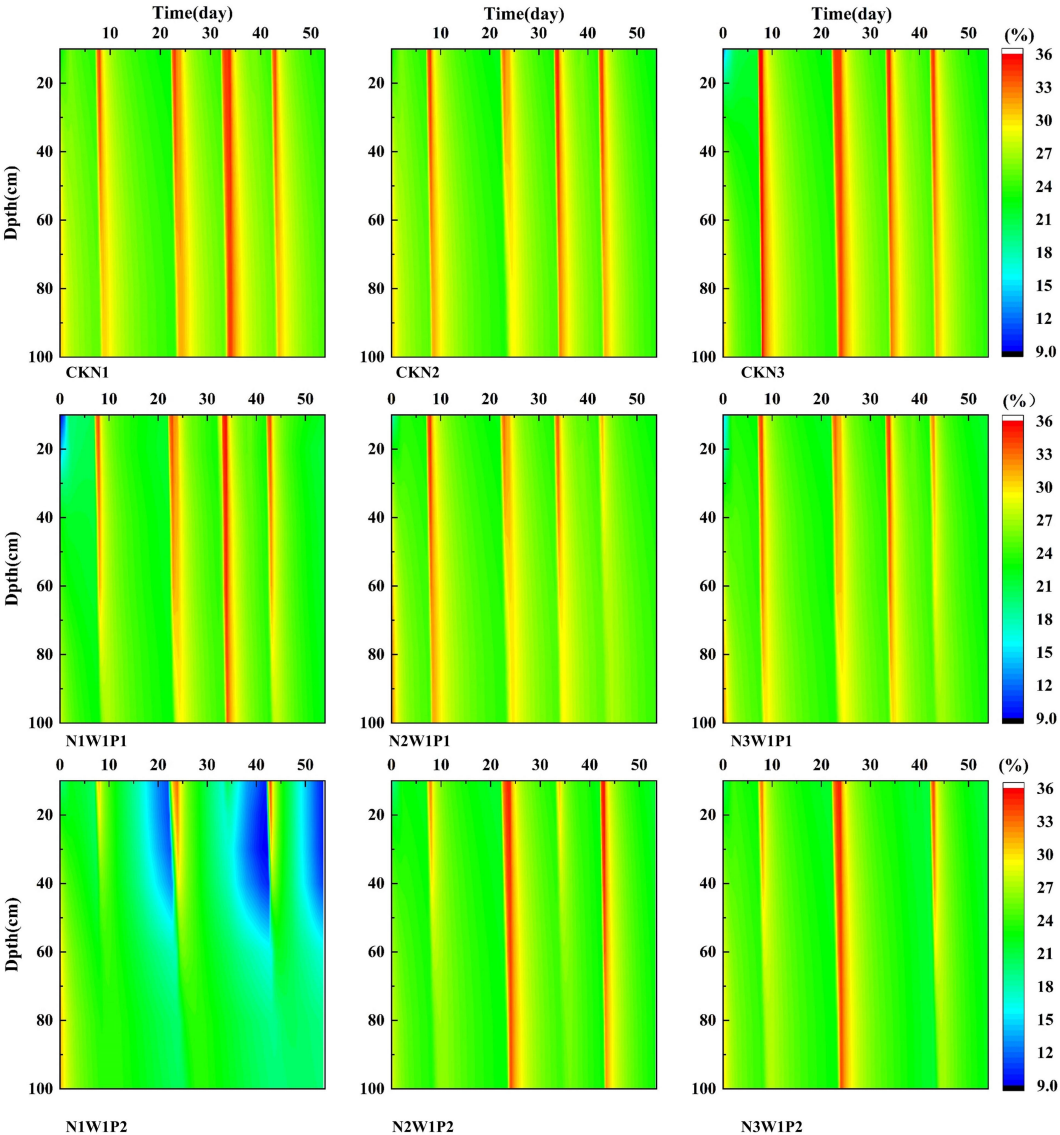
Dynamic changes in water content in soil profiles of each treatment from 2024 to 2025.

From the reviving to physiological maturity stages of winter wheat, the deep soil profile (40–100 cm) exhibited consistently higher and more stable volumetric water content compared to the surface profile (0–40 cm). The dynamic nature of surface soil moisture (0–40 cm), as depicted in **Figure 6**, was governed by a complex interplay of factors, including water extraction by crop roots, evaporation from the soil surface, irrigation applications, precipitation events during the reproductive phase, and spatial heterogeneity induced by soil texture and structure. The synergistic effect of these variables resulted in pronounced temporal fluctuations and substantial variability in the surface layer’s water content. Conversely, the soil moisture dynamics within the 40–100 cm depth were largely independent of short-term hydrological inputs. This layer was largely unaffected by light to moderate precipitation and conventional irrigation applications.

### 3.4 Transport dynamics of nitrate and ammonium in soil profiles: patterns and yield implications

#### 3.4.1 Temporal and spatial distribution of ammonium nitrogen and nitrate nitrogen

**Figure 7:**
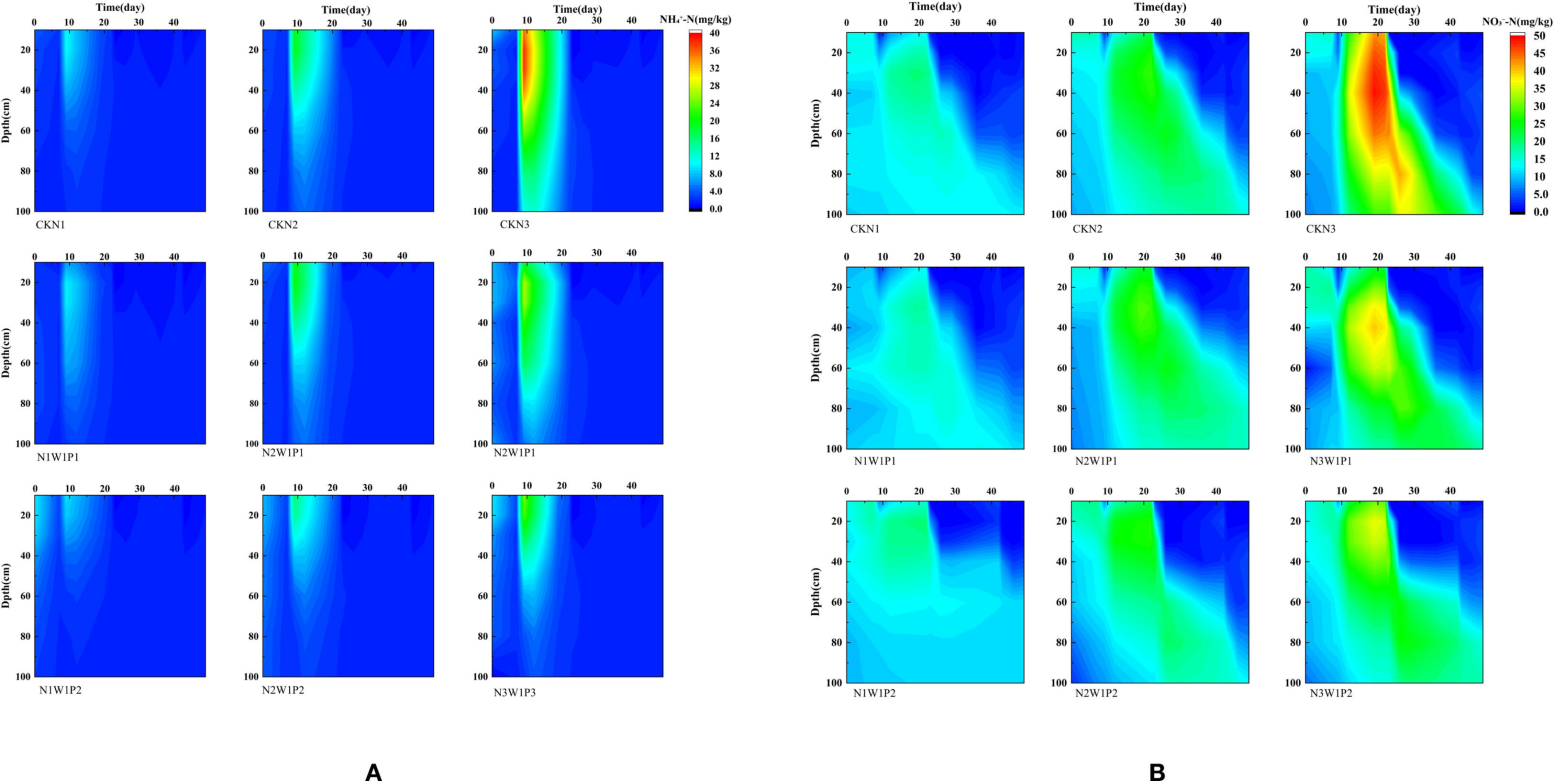
Ammonium nitrogen and nitrate nitrogen content chart. The simulation commenced from the green-up stage on March 26th, with day 7 designated as the fertilization day, day 22 as the drought rewatering day during the jointing stage, and day 42 as the drought rewatering day during the grain filling stage. **(A)** Dynamics of Ammonium Nitrogen in the soil profile from 2024 to 2025. **(B)** Dynamics of Nitrate Nitrogen in the soil profile from 2024 to 2025.

As shown in **Figure 7**, the post-fertilization distribution of mineral N exhibited pronounced spatiotemporal dependence, with contrasting dynamics for NH_4_^+^–N and NO_3_^−^–N. NH_4_^+^–N increased rapidly and accumulated in the topsoil; this accumulation was most pronounced and persistent under the high-N treatment, whereas it was weaker under the low-N treatment. In contrast, NO_3_^−^–N displayed a broader distribution and a more evident downward translocation. This divergence is primarily attributable to the low mobility of NH_4_^+^–N, which is readily retained by soil exchange sites and thus tends to build up in surface layers, whereas NO_3_^−^–N is highly mobile and is transported with percolating water. Compared with well-watered conditions, drought resulted in elevated NO_3_^−^–N concentrations in the surface soil during the dry period; however, following rewetting, NO_3_^−^–N leaching became markedly stronger and extended into deeper layers (40–100 cm), particularly under high N supply. This pattern likely reflects that drought suppresses vertical water movement and disrupts hydraulic connectivity, while rewetting re-establishes flow pathways and enhances flushing, thereby promoting nitrate translocation to depth.

#### 3.4.2 Impact of drought at the jointing stage on ammonium nitrogen and nitrate nitrogen

As shown in **Figure 7**, jointing-stage drought markedly altered mineral N dynamics relative to the well-watered control. During the drought period, NH_4_^+^-N tended to accumulate or decline more slowly in the upper soil (0–40 cm) under drought treatment (W1P1), whereas it decreased more clearly under CK. At the same time, NO_3_^−^-N redistribution was constrained under drought, with concentrations remaining more stable in the upper profile, while CK showed a more evident downward redistribution under sufficient moisture. After rewatering, the contrast became more pronounced. Upper-layer NH_4_^+^-N dropped sharply, and NO_3_^−^-N exhibited a clear downward transfer, with an enhanced signal extending into the deep layer (40–100 cm). This post-rewatering deep transport was stronger under higher N supply, indicating that larger surface pools generated during drought translated into greater downward displacement once water movement resumed.

These patterns reflect coupled process controls: drought suppresses vertical water flow and slows nitrification and transport, promoting near-surface retention, whereas rewatering rapidly restores hydraulic connectivity and stimulates conversion and flushing, thereby increasing the potential for deep nitrate transport during the jointing-stage rewetting window.

#### 3.4.3 Impact of drought on the filling stage on ammonium nitrogen and nitrate nitrogen

As shown in **Figure 7**, drought during the grain-filling stage exhibited a distinctly different response pattern compared to the control with sufficient water and drought during the jointing stage. During drought, mineral nitrogen tended to accumulate in the topsoil (0–40 cm), with both NH_4_^+^-N and NO_3_^−^-N concentrations higher than the control (CK) under drought conditions, and for a longer duration. In contrast, vertical nitrogen redistribution was weaker during this period, and changes in deeper soil layers (40–100 cm) were relatively smaller. After rehydration, the NH_4_^+^-N concentration in the topsoil decreased rapidly, indicating enhanced nitrogen transformation and consumption after water recovery. Simultaneously, NO_3_^−^-N showed a significant scouring response, with a sharp decrease in topsoil concentration and signs of downward migration. However, compared to rehydration at the jointing stage, nitrogen accumulation in deeper layers (40–100 cm) was less severe, while nitrate nitrogen loss from the topsoil remained significant.

These models suggest that drought during the grain-filling stage primarily promotes the retention of near-surface mineral nitrogen during the drought phase, while rehydration activates nitrogen transformation and migration. Because crop water requirements and irrigation amounts are lower during the grain-filling stage than during the jointing stage, the amount of nitrogen transported downwards to 40–100 cm after rehydration is relatively weak, although rehydration can still trigger a short-term surge in surface nitrate migration.

### 3.5 Impact of water and nitrogen coupling on winter wheat yield and its components

**Table 8** illustrates that soil water content, nitrogen application rate, and their interaction all had a significant effect on winter wheat yield and its components (p< 0.01). Among these factors, the lower limit of soil water content had a more pronounced impact on yield indicators than nitrogen application. The results show that under the same soil water content constraints, the trends for ear weight, 100-grain weight, and total yield of winter wheat were as follows: N2 > N3 > N1. As nitrogen application increased, the number of grains first increased and then decreased. At the same nitrogen level, the trends for 100-grain weight and total yield were: CK > P2 > P1. Notably, once soil moisture control and nitrogen application reached a certain threshold, further increases in irrigation re-strictions or nitrogen levels did not result in a significant improvement in winter wheat yield. The highest yield was observed in the CKN2 treatment, at 8702.10 kg·ha^−1^, while the lowest yield was recorded in the N1W1P1 treatment, at 4515.35 kg·ha^−1^.

**Table 8:** Significance analysis of yield.

Water management	Nitrogen treatment levels	Time	Wheat grain number	Grain weight (g)	Thousand grains weigh (g)	Wheat yield in (kg·ha^−1^)
CK	N1	2024	486.81cd	21.03d	43.2b	5551.92f
CK	N2		605.83a	32.23a	53.2a	8702.10a
CK	N3		570.86ab	28.6b	50.1a	7436.00bc
P1	N1		432.93d	17.88d	41.3b	4529.60g
P1	N2		533.07bc	26.6bc	49.9a	6419.47de
P1	N3		480.95cd	19.19d	39.9b	5014.99fg
P2	N1		477.88cd	19.88d	41.6b	5168.83fg
P2	N2		564.45ab	28.9b	51.2a	8149.80ab
P2	N3		496.84c	25.14c	50.6a	6921.88cd
CK	N1	2025	459.00c	19.80e	45.5bc	6058.83ef
CK	N2		576.00b	28.68b	53.1a	8336.32a
CK	N3		471.00c	22.80d	48.9ab	7363.79bc
P1	N1		307.50d	13.97f	45.4bc	4515.35h
P1	N2		502.50c	22.83d	50.0ab	6590.26e
P1	N3		469.50c	21.90de	46.3bc	554.81fg
P2	N1		580.50b	25.41c	43.3c	4997.32gh
P2	N2		675.00a	36.57a	52.8a	7923.46Sab
P2	N3		625.50ab	29.27b	45.1bc	7140.66d
F						
W		2024	4.44	4.14	2.39	5.73*
N			13.79**	21.88**	11.89**	17.01**
W×N			1.04	4.86**	3.07*	3.412*
W		2025	18.19**	13.16**	0.70	5.35*
N			4.45	8.16*	15.25**	19.18**
W×N			5.18**	5.03**	0.76	2.20

Different lowercase letters in the same year and the same column indicate significant differences between treatments (p< 0.05). Asterisks (*, **) indicate significance at p< 0.05, 0.01, respectively.

## 4 Discussion

### 4.1 Model validation of HYDRUS-2D

This study systematically assessed the simulation accuracy of soil water and nitrogen dynamics using the HYDRUS-2D model. The results demonstrate that the model per-formed exceptionally well in simulating ammonium nitrogen (NH_4_^+^-N) dynamics, with R^2^ values exceeding 0.85 and NSE values greater than 0.79. These findings align with research indicating that the model can accurately capture nitrogen forms, particularly ammonium nitrogen, which is primarily governed by adsorption processes in agricultural soil (Karandish and Šimůnek, 2017; Wang et al., 2023b; Xi et al., 2023). The RMSE and MAE for ammonium nitrogen were both below 3.43 and 2.33, respectively, further validating the model’s capability to reliably simulate soil adsorption-desorption dynamics (Nie et al., 2021). Additionally, parameter optimization notably enhanced the stability of the simulations, as observed by Tong et al (Tong et al., 2024) and supported by subsequent studies utilizing HYDRUS-2D’s inverse solution algorithms (Groenveld et al., 2021).

Although the simulation accuracy for nitrate nitrogen (NO_3_^−^-N) was slightly lower than that for ammonium nitrogen (R^2^ > 0.84, NSE > 0.78), it still met the research objectives. The higher RMSE and MAE for nitrate nitrogen can primarily be attributed to its strong mobility and the complexity of its multiple, coupled transformation processes (e.g., nitrification, denitrification, and root uptake) (Chen et al., 2026). This observation is consistent with findings noting that convection-dominated leaching of nitrate nitrogen is influenced by the synergistic effects of water movement, microbial activity, and root uptake, which complicate the simulation process (Chen et al., 2020). Notably, the model demonstrated high reliability in predicting nitrate nitrogen dynamics in deeper soil layers (40-100 cm) (R^2^ > 0.81), which aligns with assertions that HYDRUS is applicable for simulating solute transport in deep soil profiles (Moghimi et al., 2025; Wang et al., 2023b).

Vertical profile analysis further confirmed that the model demonstrated stable performance across the entire 0-100 cm soil profile (NSE > 0.63), with the highest accuracy observed in the root-active zone (0-40 cm) (Singh et al., 2023). This finding aligns with studies suggesting that HYDRUS, through parameter inversion, effectively captures water and nitrogen dynamics in the root zone (Amiri and Verdi, 2025) (Amiri and Verdi, 2025). Additionally, the optimization of the Feddes root water uptake model has been shown to enhance the simulation accuracy of deep soil processes (Cai et al., 2017). Furthermore, the model’s capacity to simulate non-steady-state processes, such as drought and rewatering, further validated its applicability under extreme climate conditions (Corona and Ge, 2022). Moreover, interannual differences in meteorological forcing between the calibration and validation seasons may influence model performance by altering atmospheric evaporative demand and potential transpiration, thereby affecting the magnitude and timing of root water uptake (RWU) represented as a sink term in Richards-equation–based modeling frameworks (Camporese et al., 2015). However, two limitations should be acknowledged when interpreting the results and extending the framework beyond the experimental site. First, the simulations are site-specific because soil hydraulic properties, N transformation parameters, climate forcing, and management are location-dependent; therefore, transferring the calibrated HYDRUS-2D setup to regions with contrasting soils or hydroclimates requires local parameterization and independent calibration/validation to maintain predictive reliability (Karandish and Šimůnek, 2017). Second, soil hydraulic functions were described using the standard van Genuchten–Mualem formulation, which does not explicitly represent hydraulic hysteresis between drying and wetting paths. Field observations suggest that hysteresis can be pronounced under natural forcing, and mechanistic hysteresis formulations typically require additional constraints (e.g., scanning-curve and transition information) that are difficult to robustly characterize at the plot scale (Hannes et al., 2016). Consequently, some uncertainty may remain in predicting rapid post-rewetting soil-water redistribution and the associated drainage-driven solute transport. Future work will prioritize multi-site validation across soil–climate gradients and hysteresis-enabled sensitivity tests where sufficient wetting–drying information is available.

In conclusion, the high-precision validation of the HYDRUS-2D model in this study (R^2^ > 0.84, NSE > 0.79) establishes it as a reliable tool for the subsequent analysis of nitro-gen transport mechanisms (Singh et al., 2023). The model’s parameter optimization strategy, particularly the coupled calibration of soil hydraulic properties and solute transformation parameters—offers a valuable reference for similar studies.

### 4.2 Water -nitrogen transport patterns

This study found that drought significantly inhibited the conversion of ammonium nitrogen (NH_4_^+^-N) to nitrate nitrogen (NO_3_^−^-N), while rewatering rapidly reinstated nitrification, leading to a sharp decline in ammonium nitrogen concentrations and increased nitrate nitrogen leaching. This phenomenon may be related to the response of soil microbial activity to moisture availability (Wang et al., 2023a). During the drought period (50-60% of field capacity), the activity of ammonia-oxidizing bacteria (AOB) and ammonia-oxidizing archaea (AOA) was constrained, causing ammonium nitrogen to accumulate in the surface layer. After rewatering, improved oxygen diffusion in soil pore water led to a surge in AOB/AOA activity, driving rapid ammonium nitrogen nitrification (Lv et al., 2025; Wang et al., 2022). However, rewatering also triggered deep leaching of nitrate nitrogen, especially during the jointing stage, which was linked to the high water-saturated hydraulic conductivity of the soil during this stage and the incomplete recovery of root uptake (Hao et al., 2022). The risk of leaching during the filling stage was lower, likely due to reduced crop water demand, more precise rewatering, and enhanced root ability to retain nitrate nitrogen at maturity (Ru et al., 2025).

The differential effects of drought and rewatering during the jointing and filling stages highlight the regulatory role of crop growth stages in the nitrogen cycling of the soil-crop system (Duma et al., 2022). The jointing stage is a critical period of water demand for wheat, where drought stress impairs the crop’s nitrogen uptake capacity. Fol-lowing rewatering, an excessive absorption effect occurs, significantly elevating the risk of nitrate nitrogen leaching (Rupp et al., 2021). In contrast, high-temperature drought during the filling stage more severely suppressed nitrification, with ammonium nitrogen accumulation reaching only 50% of that observed during the jointing stage. However, microbial activity recovered more slowly after rewatering due to the suppression of enzyme activity by high temperatures. Additionally, the crop’s reduced nitrogen demand during the filling stage made nitrate nitrogen leaching more manageable (Liu et al., 2022). These findings support the “growth-stage-specific water management” strategy, where precise water control during the jointing stage is essential to mitigate nitrogen loss after rewatering, while moderate drought during the filling stage can help conserve water and protect nitrogen.

High nitrogen application (300 kg/ha) under drought-rewatering conditions exacerbated nitrate nitrogen leaching, with deep-layer concentrations increasing by 40-60%, thereby confirming the “high nitrogen-high water-high loss” vicious cycle (Zhao et al., 2025). The medium nitrogen treatment (200 kg/ha) demonstrated improved nitrogen conversion efficiency (ammonium nitrogen decrease of 25-35%) and better control of leaching (deep-layer increase of 15-25%) following rewatering, aligning with the “reduced input, increased efficiency” fertilization concept (Luo et al., 2022). Notably, although the low nitrogen treatment (100 kg/ha) showed a lower risk of leaching, the insufficient accumulation of ammonium nitrogen during the drought period (peak<15 mg/kg) may hinder crop growth (Li et al., 2022). Therefore, in drought-prone regions, it is recommended to apply medium nitrogen levels (200 kg/ha) combined with small, frequent irrigation during the rewatering period to balance crop requirements and minimize environmental impact.

### 4.3 Impact of water and nitrogen regulation on winter wheat yield

Irrigation and nitrogen application are crucial factors that regulate crop growth (Abdelrhman et al., 2025). The proper balance of water and nitrogen not only enhances nutrient uptake by roots and boosts yield but also reduces agricultural non-point source pollution. This study demonstrates that the synergistic interaction between water and nitrogen significantly affects crop productivity (Li et al., 2022; Si et al., 2020). When the water level increased from W1 to CK, coupled with the N2 nitrogen application rate, wheat yield increased significantly by 31.58%, aligning with the findings of in the North China Plain (Xu et al., 2018). This phenomenon can be attributed to physiological mechanisms: moderate increases in irrigation alleviate drought stress and prevent physiological suppressions due to reduced water potential in the root zone, including decreased stomatal conductance, reduced transpiration, and the corresponding decrease in nitrogen uptake. This supports normal photosynthesis and dry matter accumulation.

In terms of nitrogen management, the study revealed a typical “threshold effect”: from treatments N1 to N3, yield initially increased and then decreased with increasing nitrogen application. Specifically, treatment N2 resulted in a 63.47% yield increase compared to N1, whereas excessive nitrogen application (N3) reduced the yield increase to only 19.38% (Mon et al., 2016; Si et al., 2020) (Mon et al., 2016; Si et al., 2020). This nonlinear response can be attributed to the underlying physiological mechanism: excessive nitrogen (N3) disrupts rhizosphere osmotic potential, thereby im-pairing root water uptake and ultimately weakening nutrient translocation capacity by reducing transpiration flux intensity—closely related to the fact that approximately 50% of nutrient elements need to be translocated to reproductive organs during fruit development (Duan et al., 2019). Notably, excessive nitrogen uptake also leads to vigorous vegetative growth, causing inefficient partitioning of photosynthetic products into vegetative organs. This may be a key reason why the fruit yield in treatment N3 decreased by 19.38% compared to N2.

Therefore, in drought-prone regions, it is recommended to apply medium nitrogen levels (200 kg/ha) combined with small, frequent irrigation during the rewatering period to balance crop requirements and minimize environmental impact (Wang et al., 2017).

## 5 Conclusions

This study used a controlled winter wheat field experiment combined with HYDRUS-2D model simulations to quantify how drought-rehydration cycles and nitrogen supply at different growth stages (jointing and grain-filling stages) regulate the redistribution, transformation, and leaching risk of NH_4_^+^-N and NO_3_^−^-N in the soil, and how these processes affect yield formation. The results showed that: (1) The HYDRUS-2D model accurately reproduced the observed dynamic changes in soil moisture and inorganic nitrogen, indicating that the calibrated model framework is suitable for diagnosing water-nitrogen coupling processes under unsteady conditions, and can be used to evaluate management programs after obtaining parameterized and validation data at specific locations.(2) The timing of drought had a significant, growth-stage-related controlling effect on nitrogen fate: drought at the jointing stage had a greater impact on nitrification, increasing the likelihood of NO_3_^−^-N migrating downwards after rehydration; drought at the grain-filling stage had a relatively smaller impact, and the risk of leaching after rehydration was also lower.(3) There was a significant interaction between water and nitrogen on grain yield, and the combination of the well-watered treatment (CK) with the moderate N rate (N2, 200 kg ha^−1^) produced the highest yield. After drought, re-irrigation should be guided by a repeatable soil-moisture threshold; maintaining soil moisture at approximately 80–85% of field capacity (FC) can relieve crop water stress while avoiding excessive refilling that increases deep percolation and thereby promotes downward NO3−-N leaching.

We note two limitations when extending the results beyond the experimental site: the van Genuchten hydraulic formula used here neglects hysteresis in the wet-dry cycle, and the calibration parameters are specific to local soil and climatic conditions. Future work should incorporate hysteresis soil hydraulic behavior and multi-site calibration/validation across contrasting soil and hydroclimatic conditions to enhance regional transferability and management universality.

## Data Availability

The raw data supporting the conclusions of this article will be made available by the authors, without undue reservation.
